# Advances in artificial muscles: A brief literature and patent review

**DOI:** 10.3389/fbioe.2023.1083857

**Published:** 2023-01-19

**Authors:** Yuan Jing, Fangfang Su, Xiaona Yu, Hui Fang, Yuehua Wan

**Affiliations:** ^1^ Periodicals Agency, Zhejiang Sci-Tech University, Hangzhou, China; ^2^ School of Economics and Management, Zhejiang Sci-Tech University, Hangzhou, China; ^3^ Library, Zhejiang University of Technology, Hangzhou, China

**Keywords:** artificial muscle, bibliometric analysis, visualized analysis, patent, scientific and technological innovation

## Abstract

**Background:** Artificial muscles are an active research area now.

**Methods:** A bibliometric analysis was performed to evaluate the development of artificial muscles based on research papers and patents. A detailed overview of artificial muscles’ scientific and technological innovation was presented from aspects of productive countries/regions, institutions, journals, researchers, highly cited papers, and emerging topics.

**Results:** 1,743 papers and 1,925 patents were identified after retrieval in Science Citation Index-Expanded (SCI-E) and Derwent Innovations Index (DII). The results show that China, the United States, and Japan are leading in the scientific and technological innovation of artificial muscles. The University of Wollongong has the most publications and Spinks is the most productive author in artificial muscle research. *Smart Materials and Structures* is the journal most productive in this field. Materials science, mechanical and automation, and robotics are the three fields related to artificial muscles most. Types of artificial muscles like pneumatic artificial muscles (PAMs) and dielectric elastomer actuator (DEA) are maturing. Shape memory alloy (SMA), carbon nanotubes (CNTs), graphene, and other novel materials have shown promising applications in this field.

**Conclusion:** Along with the development of new materials and processes, researchers are paying more attention to the performance improvement and cost reduction of artificial muscles.

## Graphical Abstract

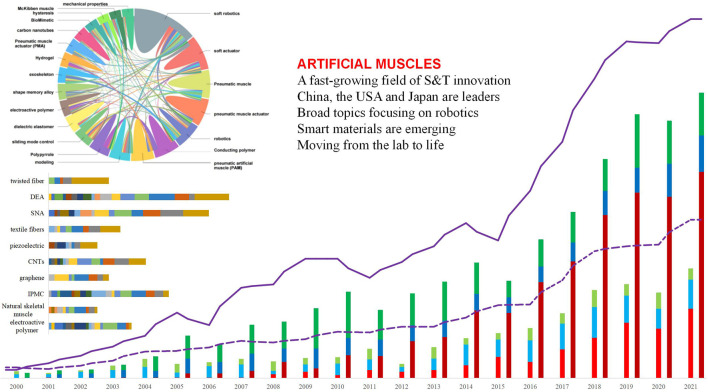

## 1 Introduction

Artificial muscle is a generic term for a class of materials and devices that can reversibly contract, expand, or rotate within one component due to an external stimulus (Mirvakili and Hunter, 2018). Due to external stimuli (voltage (Frank et al., 2022), current (Hao et al., 2019), temperature (Tada and Yoshida, 2021), pressure (Nuchkrua and Leephakpreeda, 2022), light (Li et al., 2020a; Yu et al., 2022), humidity (Zhou et al., 2018), etc.), artificial muscles can be deformed by changes in their own structure to produce the three basic actions of reversible expansion (Wang et al., 2021a), rotation (Wang H Q et al., 2020), and retraction (Oh et al., 2022). Due to the advantages of versatility, usability and high power-to-weight ratio compared to conventional motor drives (Wang et al., 2021b), artificial muscles have shown great potential for applications (Phan et al., 2021; Zakeri and Zakeri, 2022).

The study of artificial muscles emerged from intensive explorations on natural muscle and the maturation of bionic technology. The natural muscle is a soft biological actuator with excellent drive capability, compliance and self-repair ability. Drawing on the principles of natural muscle, researchers have developed film artificial muscles (Chang et al., 2021) and fiber artificial muscles (Zhang et al., 2022a) according to their macroscopic presentation. Graphene fibers (Wang R et al., 2019), carbon nanotube fibers (Jang et al., 2020), nylon fibers (Saharan et al., 2020), spandex fibers (Zhang et al., 2022b) and other artificial fibers, as well as silk (Lin et al., 2020), cotton thread (Li Y et al., 2020), and other natural fibers that constitute artificial muscles are closer to biological muscle in many properties, with good prospects for development, and have become the major direction of scientific and technological innovation of artificial muscles. After decades of development, artificial muscles have come to play a pivotal role in robotics (Dragone et al., 2022; Khalid et al., 2022), artificial limbs (Chen et al., 2020), exoskeletons (Kimura S et al., 2021) and other fields. A large number of applications in various fields have been developed, such as soft robotic swimmer (Lin et al., 2019), microhydraulic actuation for surgical instruments (Moers et al., 2012), micro flying robots (Xu et al., 2020), switch (Leng et al., 2021a), bowel peristaltic pump (Nakamura, 2022), and artificial insect (Yang et al., 2020). With the further integration with material science (Bastola and Hossain, 2021), mechanical engineering and automatic, chemical engineering, robotics and other disciplines, artificial muscles will be more widely adopted in medical (Aiyuan et al., 2021; Yarali et al., 2022), industry, military (Khan et al., 2019) and daily life (Paskett et al., 2021), and will make greater contributions to human beings.

Common review methods include systematic literature review, bibliometric methods, and meta-analysis methods. A systematic literature review can provide an in-depth and systematic review of a topic but require high academic credentials and usually only cover a small amount of literature (Snyder, 2019). Meta-analysis can extract data from a large amount of literature for a combined study of quantitative analysis and qualitative analysis on a topic (Junni et al., 2013). Bibliometric can analyze the overview of research on a topic by literature based on literature components like title, abstract, keywords, etc. The development of computer-aided analysis software makes it easy to be scaled from the micro to macro-level (Porter et al., 2019a). These three methods are not orthogonal and which one to use depends on the goals of the review and the magnitude and nature of the literature being reviewed (Donthu et al., 2021). As a knowledge-generating system that can analyze research activities and figure out future research directions in different fields based on thousands of documents (Xiang et al., 2022), bibliometric method is suitable for this study.

Bibliometrics was defined as “the application of mathematics and statistical methods to books and other media of communication” (OECD, 2022). The development of bibliometric methods can be traced back to (Lotka, 2022). Pareto published the first paper on bibliometrics (Lotka, 1926). In the following decades, Zipf, (1932), Bradford (Brookes and Bradford, 1985), Price (de Solla Price, 1974), et al. developed new bibliometric methods. Nowadays, bibliometric methodology has become an important method for review studies. It’s used in quantitative research in many fields such as materials science (Porter et al., 2019b), mechanical engineering (Li et al., 2020b; Wang et al., 2022), computer science (Darko et al., 2020; Bai et al., 2021), industry economy (Muhuri et al., 2019; Zhang et al., 2021), economics (Rousseau and Rousseau, 2021), environmental sciences (Jin et al., 2023), education (Villa et al., 2019), psychology (Tam and Milfont, 2020), public policy (Ruzhong et al., 2022; Solomon et al., 2019), and public health (Fang et al., 2021; Fang et al., 2022; He et al., 2022).

To all our knowledge, this paper is the first one to use bibliometric methods in the scientific and technological innovation of artificial muscles. Bao et al. (2018) published a bibliometric paper on soft robotics in 2018. A data-driven review is presented by Jumet et al. (2022) that addresses the recent surge of research of soft robot. But their research target and approach are different from our paper.

## 2 Methods

Our study consists of three steps. Firstly, subject, scope and objectives of the study were defined. Secondly, research data were collected. Finally, a bibliometric analysis was conducted and the results were reported.

As the first bibliometric analysis of artificial muscles, the aims of this paper are to provide a comprehensive retrospective of the progress of artificial muscles, to demonstrate the current state of artificial muscle development and to explore the future direction of artificial muscles, so the research data should include academic papers published in international mainstream journals and patent literature registered by major institutions.

The search term “artificial muscle” or “pneumatic muscle” or “bionic arm” or “bionic muscle” or “rehabilitation manipulator” was designed to retrieve research data. Two databases, Science Citation Index-Expanded (SCI-E) and Derwent Innovations Index (DII) in Web of Science (WoS) were selected as data sources. SCI-E is one of the most recognized indexing databases in the world with more than 9,500 world-renowned journals and 52 million records. DII consists of the Derwent World Patents Index (DWPI) and the Patents Citation Index (PCI), covering 96% of the world’s patent data since 1963.1,895 papers (articles and reviews) were collected from SCI-E and a search in DII got 1,970 patents with the defined research term. The retrieval date is 20 May 2022 and the retrieval field is restricted to “topic” (the title, abstract, and keywords of papers in SCI-E and title and abstract of patents in DII). Then, we carried out extended searches with search terms such as “IPMC”, “soft actuator”, and “soft robot” and cleaned the data. After removing duplicate records and some weakly relevant records by manual scanning, a total of 1,743 papers and 1,913 patents were retained and used for the follow-up analysis.

The bibliometric method was used in the data analysis of this paper. The analyzed items include bibliometric indicators such as total publications (TP) and total citations (TC), and scientific mapping such as collaborative network maps and co-word analysis maps.

We used Derwent Data Analyzer (DDA, version 10.0 build 27330) as the data cleaning and analytical tool. Like BibExcel, BibCOMB, Loet Tools, VOSviewer, Citespace, and HistCite, DDA is a data-mining tool that converts patent data, research paper and business intelligence into visualized, actionable insight. The data of papers and patents retrieved were cleaned, organized, and presented in tables and DDA charts (Fang et al., 2022). Tables were applied to show the output, collaboration and influence of journals, and authors, as well as highly cited papers and emerging topics; a chart that is a composite of line and stacked bar charts was used to illustrate the publication trend and contributions of countries/regions; pie maps were used to demonstrate the numerical relationships and co-occurrence between institutions and terms; a bubble chart was adopted to show the development trends of topics; a cluster map was used to display the field distribution of artificial muscle research and applications.

## 3 Results

The 1,743 papers were published in 492 journals and were spread over 37 years since 1961. A total of 5,207 authors from 70 countries/regions and 1,175 institutions contributed to the research of artificial muscles. As an interdisciplinary study, the 1,743 artificial muscle research papers, including 1,654 articles and 89 reviews, came from 97 WoS categories including multidisciplinary materials science, robotics, and instruments and instrumentation, etc. An average citation of 33 per paper, 57,240 total times cited, and 66,592 cited reference build the knowledge network of artificial muscles.

There are 925 patentees and 2,965 inventors who have contributed artificial muscle patents since 1975. The 1,913 patents retrieved were from 33 countries/regions, distributed in 271 International Patent Classifications (IPC) and 216 DWPI classifications.

### 3.1 Production and countries/regions’ contributions

**FIGURE 1:**
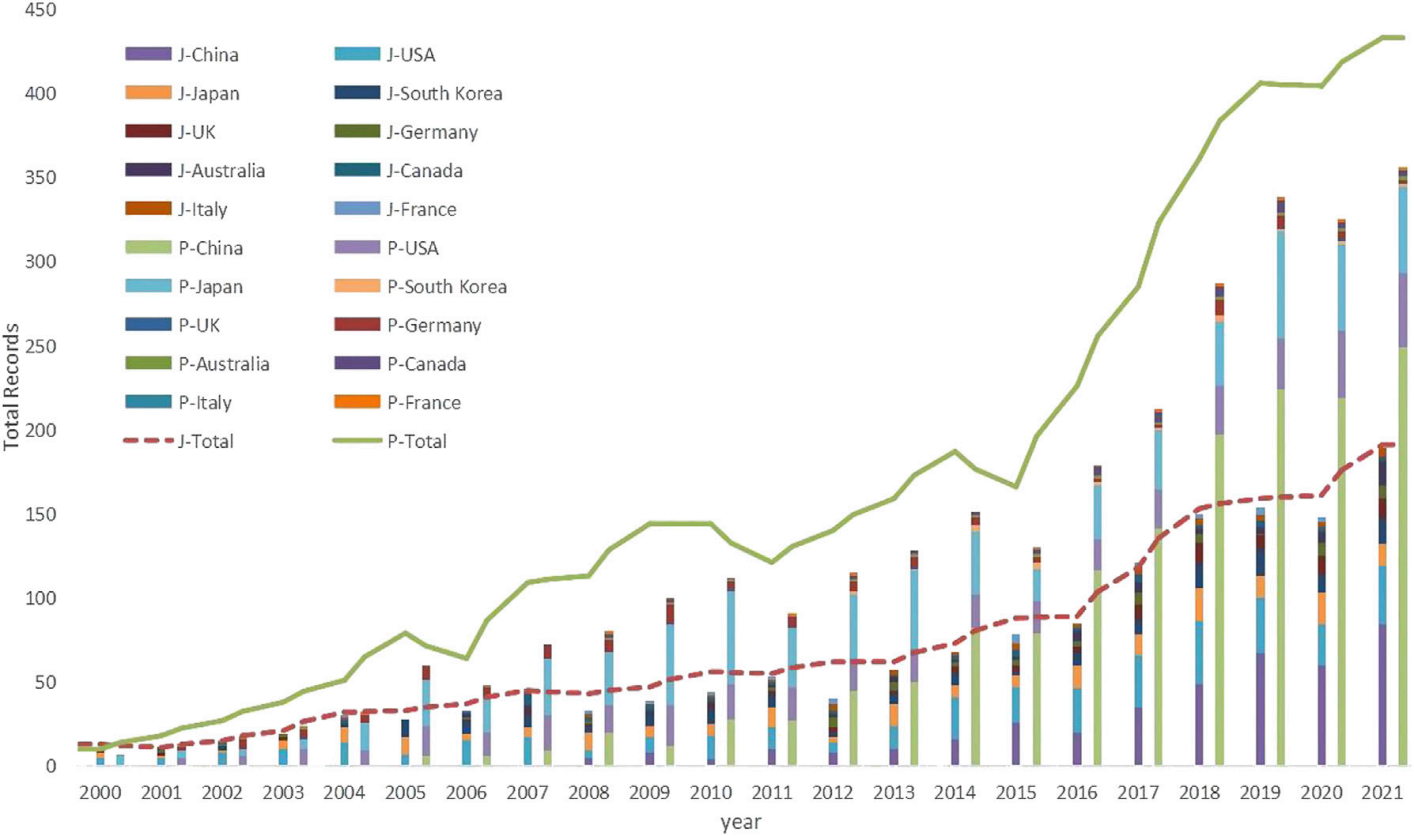
The global production of artificial muscle scientific and technological innovation and top 10 productive countries/regions contributions by year. Note(s): J-refers to the output of research papers, and P- refers to the output of technology patents.

The variation of the papers and patent output reflects the process of scientific and technological innovation on a theme. National scientific and technological innovation output is a major indicator of a country’s contribution to world science and technology. Figure 1 shows the yearly production of artificial muscles and contributions of the top 10 productive countries/regions since 2000. The paper output and patent output of the 10 countries/regions with the most papers output are presented in the form of stacked bar graphs, and the global output of papers and patents for artificial muscles are presented in the form of line graphs.

The earliest research literature on artificial muscles available in the SCI-E database is a paper published in *The Lancet* in 1961, describing a pneumatic artificial muscle invented by McKibben (Agerholm and Lord, 1961). A United States patent on fabric tendons artificial skeletal muscles (US 3882551) is the earliest artificial muscle patent in DII, disclosed in 1975.

In the decades that followed, the development of both academic research and technological applications of artificial muscles was in the gestation phase, without significant growth in related achievements. This situation did not improve until the advent of the 21st century. The number of patents and papers both exceeded 10 in 2000, and then exhibited a steady upward trend, with the growth of patents being particularly pronounced. The number of outcomes of academic research and technological applications of artificial muscles reached new heights in 2016, both exceeding or approaching 100. Since then, the growth of papers and patents on artificial muscles has become more significant, with over doubling in the 6 years from 2016 to 2021.

It is noteworthy that the top three countries/regions in terms of artificial muscle paper output, China, the United States and Japan, accounted for nearly 3/5 of the total papers, while the top 10 countries/regions in terms of output contributed nearly 4/5 of the artificial muscle papers (Table 1). The distribution of papers citation times was similar to output. The distribution of the number of patents is slightly different from that of papers. A number of artificial muscle patents are global patents or European patents. The three top-ranked countries/regions, China, Japan and the United States, have a higher percentage of patent applications than papers, reaching more than 4/5 of the total. At the same time, the specific rankings of patents and papers differ somewhat, such as the United Kingdom contributed more papers but disadvantaged in terms of patents compared to Germany.

**TABLE 1:** Contribution of the top 3 most productive countries/regions in artificial muscle research.

Year	Paper				Patent			
	Total	China	United States	Japan	Total	China	United States	Japan
2000	13	0	5	3	9	0	5	1
2001	11	1	4	1	13	0	4	5
2002	15	0	8	1	18	0	4	6
2003	21	0	10	5	24	1	6	9
2004	32	0	14	9	32	1	17	8
2005	33	1	6	10	65	6	27	18
2006	37	1	14	4	53	6	21	14
2007	45	1	16	6	75	9	34	21
2008	43	5	4	11	84	20	32	16
2009	47	8	9	7	103	12	48	24
2010	56	4	14	7	114	28	55	21
2011	55	10	13	12	98	27	35	20
2012	62	8	6	3	120	45	38	19
2013	62	10	14	13	131	50	46	20
2014	73	16	25	7	153	82	37	20
2015	88	26	21	7	134	79	19	19
2016	89	20	26	14	186	116	32	19
2017	118	35	31	12	223	141	36	23
2018	153	49	37	20	279	197	38	29
2019	159	67	33	13	321	224	64	30
2020	161	60	24	19	320	219	51	40
2021	191	84	35	13	358	249	51	44

It can also be noted from Figure 1 and Table 1 that the United States and Japan are the two veteran countries/regions in the field of artificial muscles and have been ahead of other countries/regions in both patent applications and paper publications for years. Among the emerging countries, South Korea entered this field earlier and started to catch up with the United States and Japan in 2005. China is today the leading country in artificial muscle research and application, with the number of patents and papers surpassing the United States in 2010 and 2015 (except for 2016), respectively. Other artificial muscles research forces that have emerged successively in recent years include India, Iran, and others.

### 3.2 Contribution and collaboration of institutions

**FIGURE 2:**
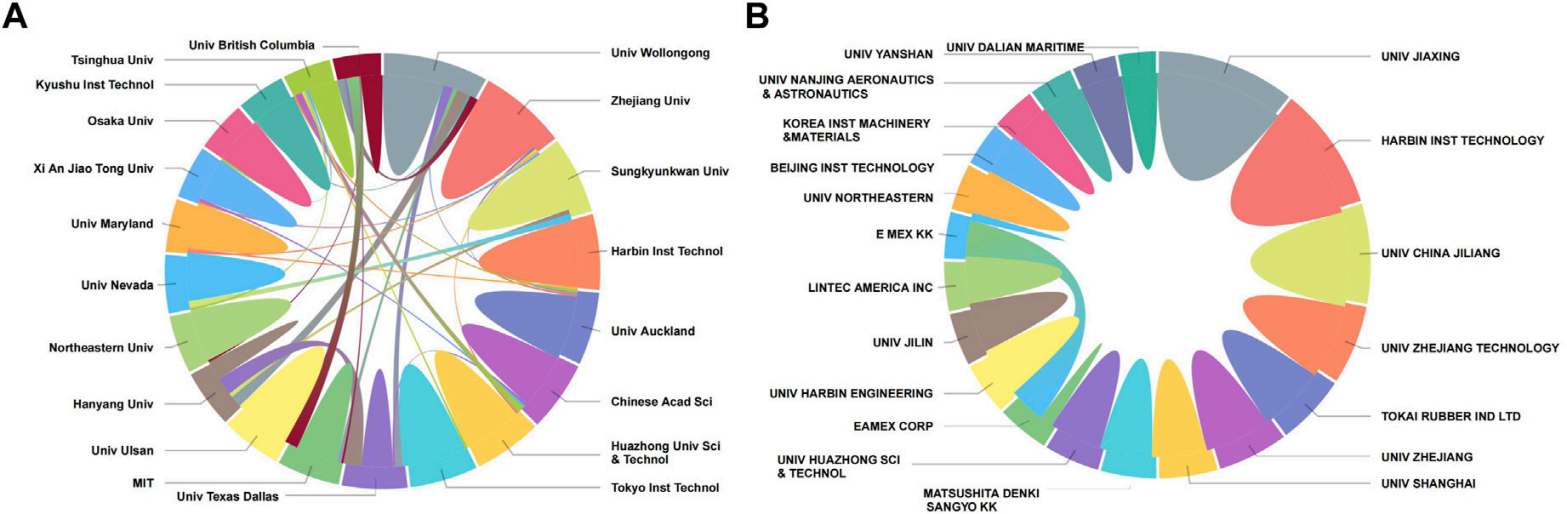
Pie map on productions and collaborations of the top 20 most productive institutions in artificial muscles **(A)** the top 20 productive institutions from a paper perspective; **(B)** the top 20 productive institutions from a patent perspective.

To show the main forces in the field of artificial muscles’ scientific and technological innovation and to reveal the collaboration between these institutions, we give information of the 20 institutions with the highest output of papers and patents of artificial muscle (Figure 2A). These 20 institutions contribute nearly one-third of the papers and papers citations on artificial muscles. The larger the proportion of an institution in the circle, the more its paper or patent output; the thicker the line between two institutions, the higher the frequency of collaboration between them. The main forces of academic research on artificial muscles are universities from different countries. Three universities, the University of Wollongong from Australia, Zhejiang University from China, and Sungkyunkwan University from South Korea, are the top three institutions in terms of paper output. Other institutions in the top ten are Harbin Institute of Technology, the University of Auckland, Chinese Academy of Sciences, Huazhong University of Science and Technology, Tokyo Institution of Technology, the University of Texas Dallas, and MIT. Among the major institutions filing artificial muscle patents, enterprises have a large share. Enterprises among the top 10 institutions are Tokai Rubber Industries Ltd., Matsushita Denki Sangyo KK, Eamex Corp, and LINTEC America Inc. Jiaxing Institute (University Jiaxing), Three Chinese universities, Harbin Institute of Technology, China Jiliang University, and Zhejiang University of Technology, rank the top 3 in the number of patent applications.

It can also be seen from Figure 2 that the frequency of academic research collaborations among the major institutions of artificial muscles is higher than that of technical applications. There are stable academic research collaboration ties among Hanyang University and the University of Texas Dallas, the University of British Columbia and MIT, the University of Wollongong and Hanyang University, and Sungkyunkwan University and the University of Nevada. Collaborative patenting among major institutions is rare, with collaboration between E MEX KK (TOYT-C) and EAMEX Corporation being one of the few cases.

With the help of temporal analysis, it can also be observed that the University of Wollongong, MIT, and Tokyo Institution of Technology were the mainstay of early artificial muscles research. Among these three institutions, the University of Wollongong has maintained the top position in the number of papers so far, MIT and Tokyo Institution of Technology have produced few papers in recent years but Tokyo Institution of Technology has had a revival in recent years. Nankai University is the representative of emerging research institutions in artificial muscles, with a rapid growth in the number of papers since 2020. Other emerging research institutions are Hohai University, Zhejiang University, etc.

Companies such as Tokai Rubber Industries Ltd., Matsushita Denki Sangyo KK, and EAMEX Corporation were among the first to layout patents on artificial muscles, but in recent years universities have begun to occupy an increasingly large share of the patent map. The current top-ranking institute in terms of the number of patents, Jiaxing University, started to lay out artificial muscle patents since 2017, and its patent applications are mainly about the design of vices, clamps, gripping heads, and manipulators’ products. It is notable that AISIN Corporation is not in the first 20 in the ranking of patent application count, but the action of manipulators’ patent layout is considerable in recent years.

### 3.3 Contribution of authors

The contribution of a researcher is usually measured by his or her publications and citation frequency. The top 20 authors in terms of number of published papers and their representative papers are presented in Table 2. Spinks from the University of Wollongong in Australia, Ahn from Ulsan University in South Korea and Kim from Nevada University in the United States ranked in the top three. In terms of frequency of citations, Baughman from the University of Texas Dallas, and Okuzaki from the University of Yamanashi occupy the top two positions, and Spinks ranks third. Spinks also has the highest h-index in the field of artificial muscles, followed by Xie from Univ Auckland, New Zealand and Kim. The high-producing scholars of artificial muscles papers are mainly from South Korea, Japan, and the United States, and there are also some high influence scholars from Australia, Belgium, New Zealand, etc.

**TABLE 2:** Contribution of the top 20 most productive authors in artificial muscle research.

Author	TP	TC	h-index	Institution	Representative paper
Spinks, Geoffrey M	27	1334	14	Univ Wollongong, Australia	Torsional Carbon Nanotube Artificial Muscles Foroughi et al. (2011)
Ahn, Kyoung Kwan	19	475	9	Univ Ulsan, South Korea	Non-linear PID control to improve the control performance of 2 axes pneumatic artificial muscle manipulator using neural network Tu and Ahn (2006)
Kim, Kwang Jin	19	797	13	Univ Nevada, United States	Recent advances in ionic polymer-metal composite actuators and their modeling and applications Jo et al. (2013)
Choi, Hyouk Ryeol	18	259	8	Sungkyunkwan Univ, South Korea	Development of enhanced synthetic elastomer for energy-efficient polymer actuators Jung et al. (2007)
Kim, Seon Jeong	18	1099	12	Hanyang Univ, South Korea	Electrochemical actuation in chitosan/polyaniline microfibers for artificial muscles fabricated using an *in situ* polymerization Ismail et al. (2008)
Kaneto, Keiichi	17	849	11	Kyushu Inst Technol, Japan	Artificial Muscle - Electromechanical Actuators Using Polyaniline Films Kaneto et al. (1995)
Wereley, Norman M	17	248	5	Univ Maryland, United States	Non-linear Control of Robotic Manipulators Driven by Pneumatic Artificial Muscles Robinson et al. (2016)
Anh, Ho Pham Huy	16	276	6	Univ Ulsan, South Korea	Hybrid control of a pneumatic artificial muscle (PAM) robot arm using an inverse NARX fuzzy model Ho and Ahn (2011)
Fernandez Otero, Toribio	16	439	8	Univ Politecn Cartagena, Spain	Sensing characteristics of a conducting polymer/hydrogel hybrid microfiber artificial muscle Ismail et al. (2011)
Hao, Lina	16	208	8	Northeastern Univ, Peoples R China	The design, hysteresis modeling and control of a novel SMA-fishing-line actuator Xiang et al. (2017)
Lefeber, Dirk	16	1263	11	Vrije Univ Brussel, Belgium	Variable impedance actuators: A review Vanderborght et al. (2013)
Nakamura, Taro	16	100	6	Chuo Univ, Japan	Joint Stiffness and Position Control of an Artificial Muscle Manipulator for Instantaneous Loads Using a Mechanical Equilibrium Model Nakamura et al. (2011)
Xie, Sheng Quan	16	744	13	Univ Auckland, New Zealand	Adaptive Impedance Control of a Robotic Orthosis for Gait Rehabilitation Hussain et al. (2013)
Baughman, Ray H	15	1792	12	Univ Texas Dallas, United States	Polymer artificial muscles Mirfakhrai et al. (2007)
Koo, Ja Choon	15	245	8	Sungkyunkwan Univ, South Korea	High performance twisted and coiled soft actuator with spandex fiber for artificial muscles Yang et al. (2017)
Oh, Il-Kwon	15	955	9	Korea Adv Inst Sci & Technol, South Korea	Durable and Water-Floatable Ionic Polymer Actuator with Hydrophobic and Asymmetrically Laser-Scribed Reduced Graphene Oxide Paper Electrodes Kim et al. (2014)
Okuzaki, Hidenori	15	1466	8	Univ Yamanashi, Japan	A Polymer Gel with Electrically Driven Motility OSADA et al. (1992)
Vanderborght, Bram	15	1262	11	Vrije Univ Brussel, Belgium	Self-healing soft pneumatic robots
Bryant, Matthew	13	155	5	N Carolina State Univ, United States	Reconsidering the McKibben muscle: Energetics, operating fluid, and bladder material Terryn et al. (2017)
Takashima, Wataru	13	497	6	Kyushu Inst Technol, Japan	Artificial muscles based on polypyrrole actuators with large strain and stress induced electrically

Note: TP = Total papers; TC = Total citations.

Other influential scholars in recent years include Baughman from the University of Texas Dallas (Mu et al., 2019; Chu et al., 2021), Mirvakili (Mirvakili and Hunter, 2017) from MIT, Shepherd (Li G R et al., 2021) from Cornell University, etc. The first study on twisted artificial muscle was jointly reported by Spinks and Baughman, then he reported a series of important studies of twisted artificial muscles (Wang R et al., 2020; Zhou D et al., 2021). Mirvakili has published papers in journals such as Advanced Materials, Science Robotics, and ACS Applied Materials & Interfaces. Shepherd’s research on topics such as 3D printing antagonistic systems of artificial muscle has been published in Science Advances, Macromolecular Rapid Communications, Bioinspiration & Biomimetics, etc.

It is noticeable that some teams focusing on artificial muscle research have emerged, such as the term of Choi, Koo, Do Nam, and Hyungpil, the term of Lefeber and Van Ham, and the term of Kaneto and Takashima.

### 3.4 An analysis of leading journals

Academic journals are the platform for scientific research achievements presentation, and high-quality research works are usually published in journals with high academic reputation. The top 20 most productive journals in artificial muscle research were listed in Table 3. Among these 20 journals, the category that appears mostly is robotics.

**TABLE 3:** Contribution of the top 20 most productive journals in artificial muscle research.

Journal	TP	TC	ACPP	h-index	Categories	If	Publisher
Smart Materials and Structures	100	2108	21.08	23	Multidisciplinary Materials Science	4.131	IOP
Sensors and Actuators A-Physical	54	1877	34.76	21	Electrical & Electronic Engineering	4.291	ELSEVIER
IEEE-ASME Transactions on Mechatronics	37	1803	48.73	18	Mechanical Engineering	5.867	IEEE
Advanced Robotics	37	507	13.7	14	Robotics	2.057	TAYLOR & FRANCIS
Actuators	35	142	4.06	7	Mechanical Engineering, Instruments & Instrumentation	2.523	MDPI
Mechatronics	33	1044	31.64	15	Mechanical Engineering, Robotics	3.379	ELSEVIER
Soft Robotics	33	887	26.88	15	Robotics	7.784	MARY ANN LIEBERT
Journal of Intelligent Material Systems and Structures	32	636	19.88	12	Multidisciplinary Materials Science	2.774	SAGE
IEEE Robotics and Automation Letters	32	174	5.44	6	Robotics	4.321	IEEE
Bioinspiration & Biomimetics	30	914	30.47	11	Biomaterials Materials Science, Robotics	2.985	IOP
Sensors and Actuators B-Chemical	27	1122	41.56	15	Electrochemistry, Instruments & Instrumentation	9.221	ELSEVIER
IEEE Access	25	128	5.12	5	Engineering Computer Science, Electrical & Electronic	3.476	IEEE
Synthetic Metals	24	1188	49.5	10	Multidisciplinary Materials Science	4	ELSEVIER
ACS Applied Materials & Interfaces	23	973	42.3	14	Multidisciplinary Materials Science	10.383	ACS
Advanced Materials	21	2734	130.19	21	Multidisciplinary Materials Science	32.086	WILEY
IEEE Transactions on Industrial Electronics	19	657	34.58	10	Engineering, Electrical & Electronic; Instruments & Instrumentation	8.162	IEEE
Applied Sciences-Basel	17	68	4	5	Engineering, Multidisciplinary; Multidisciplinary Materials Science	2.838	MDPI
Advanced Functional Materials	15	1030	68.67	7	Nanoscience & Nanotechnology; Multidisciplinary Materials Science	19.924	WILEY
Journal of Bionic Engineering	14	197	14.07	6	Engineering, Multidisciplinary; Robotics	2.995	SPRINGER
Electrochimica Acta	13	495	38.08	8	Electrochemistry	7.336	ELSEVIER

Note: TP = Total papers; TC = Total citations; ACPP = average citation per paper; IF = Impact Factor 2021.

In terms of the number of published artificial muscle papers, *Smart Materials and Structure*s, *Sensors and Actuators A-Physical*, *IEEE-ASME Transactions on Mechatronics*, *Advanced Robotics*, *and Actuators* are in the top 5. For the citation of papers, *Advanced Materials*, *Smart Materials and Structures*, *Sensors and Actuators A-Physical*, *IEEE-ASME Transactions on Mechatronics*, and *Synthetic Metals* ranked in the top 5. Different from the number ranking, *Advanced Materials* received 2,734 citations with only 21 papers, surpassing *Smart Materials and Structures*’ 2,108 citations with 100 papers. *Advanced Functional Materials* is also on the rise. Regarding average citation per paper, *Advanced Materials*, *Advanced Functional Materials*, *Synthetic Metals*, *IEEE-ASME Transactions on Mechatronics*, and *ACS Applied Materials* & *Interfaces* performed the best. For h-index, *Smart Materials and Structures*, *Advanced Materials*, *Sensors and Actuators A-Physical*, *IEEE-ASME Transactions on Mechatronics*, *Sensors and Actuators B-Chemical* are in the top 5.

If only considering the number of artificial muscle papers published in the last 3 years, *Smart Materials and Structures* is still in the first place. The top ranking journals are *Smart Materials and Structures*, *Actuators*, *IEEE Robotics and Automation Letters*, *IEEE Access*, *Sensors and Actuators A Physical*, *Soft Robotics*, *Bioinspiration Biomimetics*, *Journal of Robotics and Mechatronics*, and *Polymers*. *Physical*, *Soft Robotics*, *Bioinspiration Biomimetics*, *Journal of Robotics and Mechatronics and Polymers*, *Actuators*, *IEEE Robotics and Automation Letters*, *IEEE Access*, and *Polymers* are representatives of the emerging journals. In recent years, more papers on artificial muscles have also been published in *Science Robotics*, *Advanced Materials Technologies*, *Science Advances*, etc. *Synthetic Metals*, *Electroactive Polymerica*, *Journal of Electroanalytical Chemistry*, and *Macromolecules* have decreased the publication of artificial muscle papers in recent years.

The top 20 journals in terms of number of publications include five and four journals from Elsevier and IEEE, respectively. Five major publishers, Elsevier, IEEE, IOP, Springer, and Wiley, published more than half of the artificial muscle research papers. Elsevier ranked first with 282 papers. MDPI is the representative of emerging publishers.

### 3.5 An analysis of prominent fields

**FIGURE 3:**
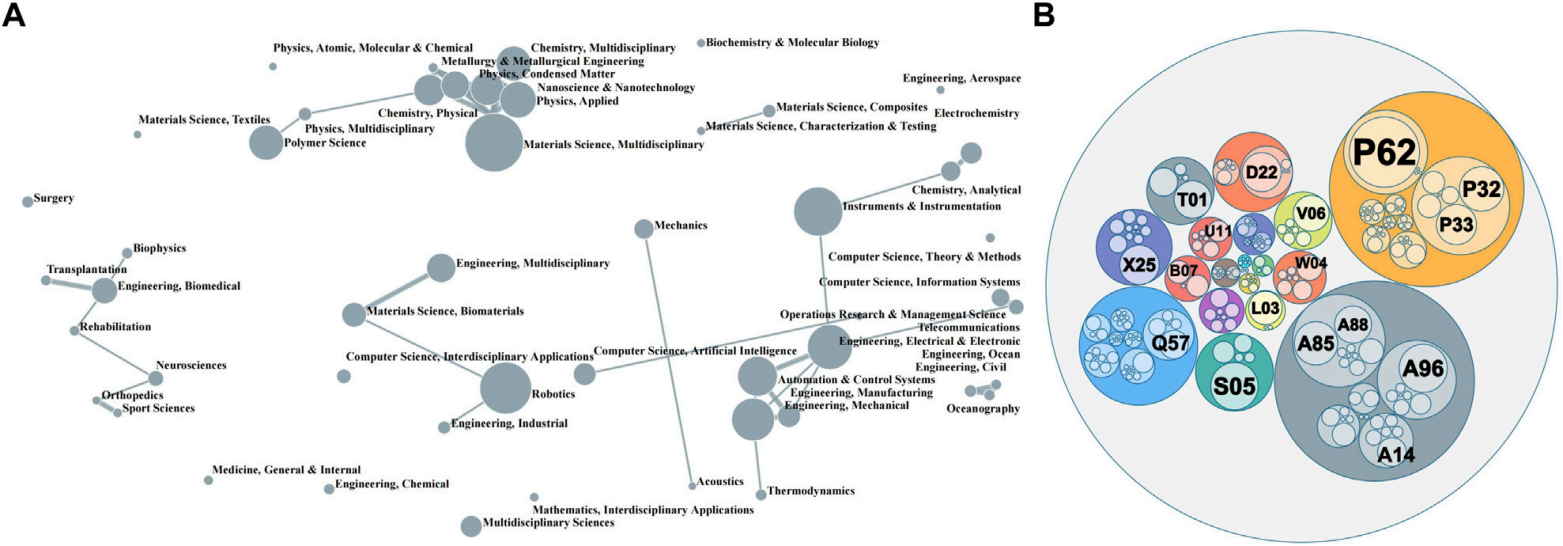
Cluster map of artificial muscle scientific and technological innovation fields **(A)** cluster of papers’ WoS categories; **(B)** cluster of patents’ DWPI categories.

As a Frontier scientific problem, artificial muscle research covers a wide range of fields. Figure 3 shows the WoS category clustering map of the artificial muscle papers and the DWPI category clustering map of the patents. The size of a dot represents the number of papers, and the thickness of the line between the dots indicates the intensity of the interaction between the two fields. Multidisciplinary materials science, instruments instrumentation and robotics are the major fields of artificial muscle research, making up more than half of the total papers. In the patent clustering circle pack diagram, the DWPI patent categories are shown in concentric circles. The large circles indicate high-level categories, and the smaller circles in the circles indicate related categories of lower levels. The size of each circle indicates the number of documents in that category.

Research on artificial muscles is concentrated in three clusters, the materials science cluster consisting of multidisciplinary materials science, applied physics, multidisciplinary chemistry, and others, the mechanical and automation cluster consisting of instruments and instrumentation, electrical and electronic engineering, mechanical engineering, automation and control systems, etc, and the robotics cluster consisting of robotics, biomaterials materials science, interdisciplinary applications computer science, and industrial engineering. There are also some more independent fields including textile, surgery, aerospace engineering, ocean engineering, civil oceanography engineering and so on.

The largest cluster of artificial muscle patents is the general engineering cluster, followed by the polymers chemical cluster and the instrumentation cluster. Among the general engineering clusters, hand tools (P62), prosthesis (P32), and medical aids (P33) appear most frequently. The instrumentation cluster is mainly focused on electrical medical equipment (S05). In addition, industrial electric equipment (X25), bandages (D22), and digital computers (T01) also have a sizeable patent portfolio.

### 3.6 Development of various kinds of artificial muscle

**FIGURE 4:**
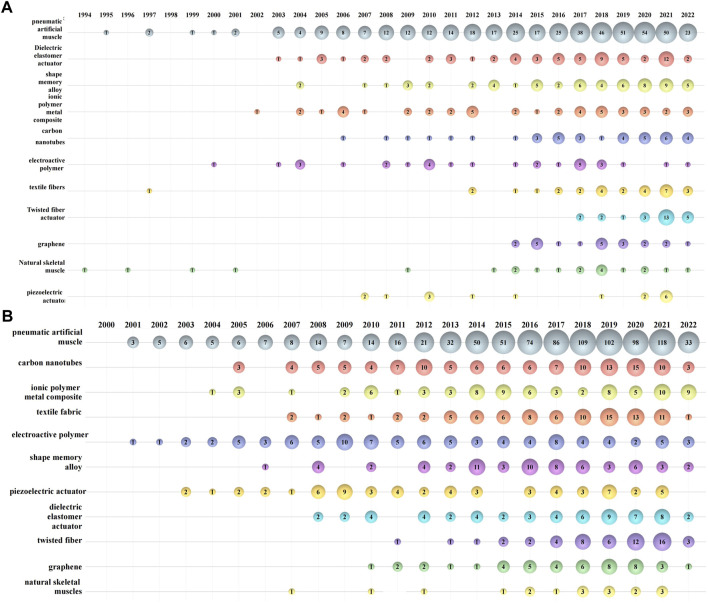
Bubble chart of various artificial muscle development **(A)** development in papers **(B)** development in patents.

Various kinds of artificial muscle have been investigated. On the basis of different stimuli, artificial muscles can be classified as electrical stimulation, photo stimulation, chemical stimulation, humidity stimulation, magnetic stimulation, pressure stimulation, etc. These artificial muscles can be classified into many types depending on the power mechanism and material. For example, electro-active polymer (EAP) can be classified to ionic EAP, electronic EAP, and electro-thermal EAP by stimulation mechanism, and nanoparticle-Based actuators are represented by actuators using new materials such as carbon nanotubes, graphene and fullerenes. Figure 4 illustrates the evolution of research and applications of various artificial muscles. Since the number of papers before 1994 and patents before 2001 are minor, the papers and patents in the figure were dated back to 1994 and 2001, respectively. The artificial muscles selected in this paper are PAMs, DEAs, SMAs, IPMCs, carbon nanotube (CNT) electroactive polymers, textile fiber based actuators, twisted fiber actuators, graphene based actuators, natural skeletal muscles, piezoelectric actuators and other artificial muscles.

PAMs is the major artificial muscle research and application topic for its high compliance, safety, and force (Abu Mohareb et al., 2021; Liang et al., 2022a), with the same long history as natural skeletal muscle. Since Pelrine et al. investigated the large strains, fracture toughness, and fast response of acrylic and silicone elastomers compared to a natural muscle in 2000 (Pelrine et al., 2000), DEA related papers have been studied extensively, but the technology development is rare. SMA artificial muscles are a kind of artificial muscles based on the shape memory effect of alloys originating from a phase transition due to temperature variation (Park et al., 2020), and researchers are working to overcome the limitation of low working frequencies impose on its application (Choi et al., 2022). CNTs have been used widely in the field of artificial muscles in recent years due to the excellent microwave thermal effect and mechanical properties (Aziz et al., 2021), especially in the area of patent applications. Twisted fiber actuator and graphene are the popular emerging materials in recent years (Ghosh and Karak, 2021; Kimura D et al., 2021), and the related research and development is hot. Unlike natural skeletal muscle, about which the scientific and technological innovation scale is getting smaller, textile fibers have been growing in patents in the field of artificial muscles. Piezoelectric artificial muscles have relatively few papers and many patents but their high power density and simple structure (Kanik et al., 2014; Mokhtari et al., 2021) make them a possible hot spot for future artificial muscle research and development.

### 3.7 A discussion of most discussed terms

**FIGURE 5:**
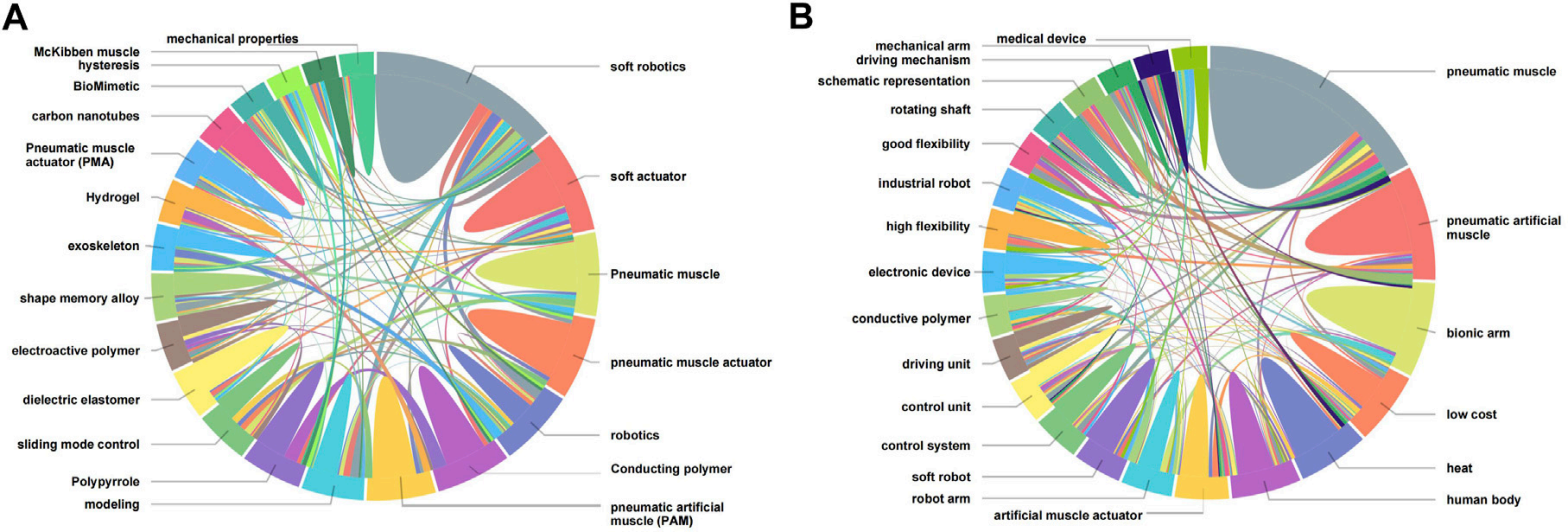
Pie map of the top 20 terms of artificial muscle **(A)** author keywords of papers; **(B)** abstract NLP of patents.

The top 20 terms of the paper and patent outputs in the field of artificial muscles are shown in Figure 5A, correspondingly. In them, the terms of papers come from the cleaned author keywords of papers and the terms of patents come from the results of natural language processing (NLP) of patent abstracts. The more a topic occupies in the circle, the more its paper or patent output; the thicker the line between two topics, the higher the frequency of their common occurrence.

As seen in Figure 5A, the academic research on artificial muscles focuses on the research and development of novel materials. Hot research terms include soft robotics, soft actuator, pneumatic muscle, pneumatic muscle actuator, robotics, conducting polymer, PAMs, modeling muscles, sliding mode control, DEA, electroactive polymer, modeling muscles, sliding mode control, dielectric elastomer, electroactive polymer, etc. Due to the strong correlation between artificial muscle research and soft robotic research, soft robotic research has connected most of the artificial muscle research terms. PAMs, as the most commonly used artificial muscle type, has also led to the development of similar term research.

As far as the scientific and technological innovation of artificial muscles goes, researchers have focused more on the performance and functional structure of artificial muscle products in the patent application process. PAMs, bionic arm, low cost, heat human, body artificial muscle actuator, robot arm, soft robot control system, control unit, driving unit, conductive polymer electronic device, high flexibility, industrial robot, and good flexibility are the most involved terms in artificial muscles patents. The pursuit of higher performance, more application scenarios, and lower production costs is the main reason for the discrepancy between the artificial muscle technological innovation terms and the academic research terms.

### 3.8 An analysis of scientific and technological innovation frontiers

To reveal the scientific and technological innovation frontiers of artificial muscles, we analyzed the ESI highly cited papers, highly cited patents, hot paper, and emerging terms. The ESI highly cited papers are the top 1% cited papers in the research field published in the past 10 years. Usually, a paper selected as an ESI highly cited paper indicates that its research direction is the current focus of the academic community, and the ESI highly cited papers can indicate the focus of a research theme. The ESI highly cited papers on artificial muscle research since 2012 is shown in Table 4.

**TABLE 4:** ESI highly cited papers of artificial muscle research.

Paper	Source title	Institution	TC	DT
Hines et al. (2017)	Advanced Materials	Max Planck Inst Intelligent Syst	619	Review
Zang et al. (2013)	Nature Materials	Duke Univ	617	Article
Harada et al. (2014)	Accounts of Chemical Research	Osaka Univ	599	Review
Vanderborght et al. (2013)	Robotics and Autonomous Systems	Univ Pisa	590	Review
Takashima et al. (2012)	Nature Communications	Osaka Univ	463	Article
Anderson et al. (2012)	Journal of Applied Physics	Univ Auckland	454	Review
Acome et al. (2018)	Science	Univ Colorado	403	Article
Mirvakili and Hunter (2018)	Advanced Materials	MIT	386	Review
Bruns and Stoddart (2014)	Accounts of Chemical Research	Northwestern Univ	375	Review
Cianchetti et al. (2018)	Nature Reviews Materials	Scuola Super Sant Anna	370	Review
Park et al. (2014)	Bioinspiration & Biomimetics	Carnegie Mellon Univ	290	Article
Yang et al. (2015)	Nano Letters	Chinese Acad Sci	284	Article
Li et al. (2017)	Proceedings of the National Academy of Sciences of the United States of America	Harvard Univ	270	Article
Romasanta et al. (2015)	Progress in Polymer Science	CSIC	254	Review
Madsen et al. (2016)	Macromolecular Rapid Communications	Tech Univ Denmark	249	Review
Gu et al. (2018)	Science Robotics	Shanghai Jiao Tong Univ	218	Article
Wang et al. (2018)	Advanced Materials	Univ Houston	208	Article
Chen et al. (2018)	Nature Chemistry	Univ Groningen	204	Article
Terryn et al. (2017)	Science Robotics	VUB	200	Article
Lee et al. (2018)	Science Advances	Seoul Natl Univ	199	Article
Kanik et al. (2019)	Science	MIT	148	Article
Qin et al. (2019)	Nature Communications	Hefei Univ Technol	148	Article
McCracken et al. (2020)	Advanced Materials	Univ Colorado	107	Review
Zare et al. (2019)	Chemical Engineering Journal	Natl Univ Singapore	84	Review
Umrao et al. (2019)	Science Robotics	Korea Adv Inst Sci & Technol	82	Article
Sun et al. (2020)	IEEE Transactions on Industrial Informatics	Nankai Univ	80	Article
Chen et al. (2021)	Angewandte Chemie-International Edition	Univ Groningen	25	Review

Note: TC = total citations; DT = document type.

Reviews can provide a systematic overview of the progress of a research direction, and articles can report the latest original findings in a timely manner. The 27 highly cited papers, especially the 15 articles, revealed the frontiers of artificial muscle research. In Takashima et al. 2012 designed a photoresponsive supramolecular actuator by integrating host-guest interactions and photoswitching ability in a hydrogel. Zang et al. reported an approach to reversibly control the crumpling and unfolding of large-area graphene sheets used as artificial muscles actuators in 2013. Park et al. designed a wearable robotic device powered by PAMs in ankle-foot rehabilitation in 2014. In Yang et al. (2015) studied the optical, magnetic, and electrical properties of ReSe2, which enabled an application of artificial muscles actuators. Li et al. (2017) reported an artificial muscle in 2017, which can be driven by fluids at negative pressures. Diels-Alder polymers were used to develop artificial muscle applications of self-healing pneumatic actuators by Terryn et al. Acome et al. described a class of hydraulically amplified self-healing electrostatic actuators in 2018. Gu et al. reported a soft wall-climbing robot based on muscle-like actuators. A soft robot that senses environment and adaptively performs soft crawling with artificial muscles is reported by Wang et al. (2018). Lee et al. reported an organic optoelectronic sensorimotor synapse for light-interactive actuation of an artificial muscle actuator. In Kanik et al. (2019) applied a high-throughput iterative fiber-drawing technique to create strain-programmable artificial muscles. Qin et al. described a general *in situ* polymerization strategy for the fabrication of anisotropic hydrogels. Umrao et al. reported an MXene artificial muscle with ultrafast rise time. In Sun et al. (2020) reported a new adaptive control method of PAMs systems.

Besides the ESI Highly Cited Papers, there are also papers published over 10 years ago that are still frequently cited by researchers in recent years. A paper published in 1996 tested the McKibben artificial muscle pneumatic actuator first developed in the 1950s, which became a classic work of McKibben artificial muscle research, and is still of great reference value to researchers today (Chou and Hannaford, 1996). The principles involved in a 2004 paper by Madden et al. (2004) that reviewed the physical principles of artificial muscle technology are still widely used today. The DEA research progress reported by Brochu et al., in 2010 is still of great reference value to researchers today (Brochu and Pei, 2010) and a chemomechanical system based on a synthetic polymer gel was reported by OSADA et al. (1992) opening a new window of artificial muscle R&D. The same goes for Mirfakhrai’s research in 2007 on the mechanisms and performance related to polymer artificial muscle technology (Mirfakhrai et al., 2007), Pelrine’s paper on elastomeric dielectric materials with compliant electrodes (Pelrine et al., 2000), and Thomsen’s work on liquid crystal elastomers with mechanical properties of a muscle (Thomsen et al., 2001). It is notable that the research themes of high-level journal papers are more likely to be of sustained interest to researchers. For instance, a 2005 research paper published in Nature on motors capable of inducing mechanical motion (van Delden et al., 2005) and a paper on torsional CNTs artificial muscles in Science (Foroughi et al., 2011) are still frequently cited today.

Unlike ESI highly cited papers, ESI hot papers reflect the latest Frontier issues in the field. ESI hot papers are papers published in the past 2 years that have received a number of citations in the most recent 3-month period that places them in the top .1% of papers in the same field. Hot papers generally conduct research geared toward interdisciplinary Frontier issues. Artificial muscles-related topics currently have a paper published in Nature in 2021 that was selected as an ESI hot paper, exploring the application of artificial muscles in extreme environments. The paper designs a soft robot with artificial muscles, power and control electronics distributed on a polymer matrix that can swim freely in a deep ocean high pressure environment like a deep ocean fish (Li S et al., 2021).

When we talk about the technological innovation of artificial muscles, highly cited patent is an important reference indicator (Table 5). The industry’s most interesting artificial muscle technologies include: process for producing artificial muscles electric conductive polymers (WO2004014987, WO200158973), SNA fibre actuator (US5092901), carbon nanotube actuator (WO2005102924, WO2007033438), artificial muscle hydrogel polymer (US5100933, WO2008079440), and magnetorheological fluid in artificial muscle tissue (US6168634), artificial muscle-skeletal mechanism (US4776852), bilayered structure artificial muscles (JP2006297005). Highly cited patents for artificial muscles are mainly from two scenarios: implementation of products and improvement of technologies. The current high-priority artificial muscle products are mainly from the medicine and healthcare fields, such as wearing hand function rehabilitation training robot (CN101181176, CN101433491), flexible bladder device (US6223648), artificial sphincter (US2003212306, US2005004425), apparatus for augmenting near vision accommodation (US2003028248), cardiac repair artificial muscle sheath (US4176411), implantable cardiac defibrillation devices (US5578069), and artificial anatomic lumen structure model (WO2006083963).

**TABLE 5:** The top 20 highly cited patents of artificial muscles.

Title	Institution(s)	Patent(s)	TC	Year
Process for producing electric conductive polymers for use for functional parts in driving devices and for artificial muscles comprises electrolytic polymerization of pyrrole in an electrolytic solution containing an organic compound	EAMEX CORP, et al.	WO2004014987, JP2004162035, AU2003254887, et al.	459	2004
Artificial sphincter for treating fecal incontinence and urinary incontinence, comprises cuff comprising electroactive polymer actuator(s), and control unit	BANIK M S, et al.	US2003212306, WO2003094800, EP1503703, et al.	131	2004
Artificial anatomic model for testing device for invasive transmission, comprises luminal structure comprising analog material simulating predetermined physical characteristics of inner luminal surface, and artificial support tissue	SAKEZLES C	WO2006083963-A2, US7272766-B2, EP1848332-A2, et al.	113	2006
Electroactive polymer device for, e.g. motor or generator, includes electroactive polymer(s) having active area which comprises electrodes	SRI INT	WO200158973-A2, AU200149058-A, US2002008445-A1, et al.	94	2001
Ionised crosslinked polyacrylamide gel compsns. - when exposed to e.g. electromagnetic radiation, exhibit drastic volume changes in response to minor changes in e.g. pH	MASSACHUSETTS INST TECHNOLOGY	US5100933, US35068-E	85	1992
Artificial muscle tissue for robotics, cybernetics and artificial prostheses, has magnetorheological fluid which flows in linearity stiff and radially compliant fiber interior through valves	SCHMITZ G W	US6168634	82	2001
Aggregate of carbon-based fine structure used for electronic device, is obtained by orientating several carbon-type fine structures in same direction, and gathering fine structures along orientation direction	JAPAN SCI&TECHNOLOGY AGENCY, et al.	WO2005102924, EP1777195, KR2007027549, et al.	79	2005
Implantable deployable defibrillation electrode mechanism - employing artificial muscle elements to increase electrode surface geometry to reduce shunting of current through blood	VNETRITEX INC.	US5578069	72	1997
Artificial skeletal mechanism for robotic actuator - has central rigid inverted T = shaped link and paired resilient tubular actuators on either side	BUBIC F R	US4776852, CA1260986	66	1988
Flexible actuator assembly for biorobotics, has sliding seal formed between bladder device and tendon component to seal chamber during reciprocating movement between extended and retracted condition	ERICKSON J R	US6223648	62	2001
Elastic epoxy hydrogel polymer composition used as oxidizing coating, and ion exchange membrane for actuator used for pump for drug delivery device, contains reaction product of polyether amine, and polyglycidyl ether	MEDIPACS INC., BANISTER M, et al.	WO2008079440, EP2041214, IN200900137, et al.	56	2008
Electroactive polymer transducer for use as e.g. lens actuator of camera, has caps fixed to polymer layers such that caps are less flexible than polymer layers	HEIM J, et al.	US2006208610, WO2006102273, EP1861885, et al.	55	2006
Actuator comprises modified shape memory alloy fibre - where memory alloy e.g. nickel@-titanium@ has shorter switch response useful in robotics and prosthetics	MCGILL UNIV	US5092901	55	1992
Artificial sphincter for treating e.g. fecal incontinence by implantation into patient, comprises cuff including electroactive polymer actuators, and control unit for electrically controlling electroactive polymer actuators	BANIK M S	US2005004425, US6921360	54	2005
Apparatus for augmenting near vision accommodation by contraction of the ciliary muscles of the eye by reinforcement of at least one set of zonular fibers, comprises at least one bridge affixed to the zonular fibers	SHAHINPOOR M	US2003028248, US7060094-B2	48	2004
Catalyst system, useful for selective conversion of hydrocarbons into multi-walled carbon nanotubes and hydrogen, comprises nickel, cobalt, iron and aluminum oxide complex	NANOCYL SA	WO2007033438, EP1797950, US2008206125, et al.	47	2007
Cardiac assist device using electrically stimulated artificial muscle - consists of center atrial to descending thoracic aorta shunt surrounded by electromagnetically actuated sheath rods formed of elastomer	RUNGE T M	US4176411	44	1979
Wearing hand function rehabilitation training robot for patient, has middle connecting pieces are arranged with compression springs, where ends of springs are in contact with connecting rod face stretching part into connecting piece	UNIV HUAZHONG SCI & TECHNOLOGY	CN101433491	43	2009
Wearable hand-functional rehabilitation robot for assisting patient, has pneumatic muscle whose inlet end are connected with back end of mechanical arm, and tension rod fixed with rigid string	UNIV HUAZHONG SCI & TECHNOLOGY	CN101181176, CN100594867	43	2008
Artificial muscle of bilayered structure, consists of first layer that is obtained using easy polymer gel having swelling and shrinkage property and second layer formed using electrostriction polymer with actuators	ANDO Y	JP2006297005	43	2007

Note: TC = total citations.

To help researchers identify emerging research areas worthy of attention in the field, we calculated emerging topics in artificial muscle scientific and technological innovation with DDA (Table 6). The DDA Emerging Trends Indicator tracks the evolution of subject terms based on four key factors: novelty, persistence, degree of community, and growth, demonstrating a temporal sequence of subject evolution with some predictive power (Porter et al., 2020). The Emerging Trends Indicator is calculated using words or phrases mined from the titles and abstracts of patents from the past 10 years to detect emerging subject terms based on a benchmark of the previous 3 years (Li M et al., 2021).

**TABLE 6:** Emerging trends of artificial muscle patents.

Rank	TP	Emerging trend	Score	Partial patents
1	17	Robot device	16.874	GB 2354752, IN 201941049396, JP 2022047779, JP 2022047780, JP 2022047782, US 2021370499, WO 2022054948, WO 2022054949
2	22	soft robot	10.842	CN 114055453, CN 114367960, KR 2019014346, US 11149192, US 2021175409, US 2022065271, WO 2018185183, WO 2021055063
3	11	waist joint	8.329	CN 112775953, CN 113146580, CN 114346998, CN 114346999
4	20	supply	6.486	CN 113054090, CN 113532520, JP 2021191973, US 2022015932, US 2022021314, US 7935743, WO 2016031111, WO 2022043804
5	14	fluid pressure	6.05	CN 113882025, CN 114081800, CN 114346999, CN 204698965, KR 2015029318, RU 2232862
6	29	motion	5.542	AU 2021100301, CN 206242039, CN 206811944, DE 102007053382, JP 2017079034, JP 2021078621, US 2022015977, US 6168634, WO 2020055342, WO 2021006309
7	18	knee joint	5.018	CN 204450526, CN 215081660, US 2021220205, WO 2021201604
8	8	fast response speed	4.129	CN 111658434, CN 113443037, CN 114074593, CN 114227661, US 2021180620,
9	18	electric energy	3.864	CN 113501060, CN 213546985, DE 102017210666, JP 2009273201, JP 61004731, KR 2016117658, KR 989410, US 7935743, WO 200158973, WO 2022019858

Note: TP = Total patents.

There are nine emerging topics in the field of artificial muscle patents. Soft robots and robotic devices are the main applications of artificial muscles and are receiving attention from researchers from China, India, South Korea, Japan, the United States, and other countries. As a key connector for key parts such as knee joints, waist joints, and lower limb joints, artificial muscles have lots of applications in pneumatic system-based simulated robot systems. A continuous and stable power supply is a prerequisite for artificial muscles to work, and power supply devices are a necessary panel for patent layout. In order to enable robots to effectively work in more extreme environments, researchers in China, South Korea, Russia, and other countries have carried out research and development of artificial muscles to cope with fluid pressure. Compared with biological muscle, the reaction speed of artificial muscles has always been slow, which restricts the application of artificial muscles, and researchers in China, the United States, and other countries have also been working to obtain a more rapid reaction speed of artificial muscles.

Another way to identify Frontier issues is to observe the variation of topics across time. We counted the 20 most frequently occurring terms in papers and patents from 2020 to 2021 and compared them to their frequency of occurrence during 2018–2019 (Table 7). The growth topics of artificial muscle research in the last 2 years are SMA, twisted fiber actuator, CNTs, piezoelectric actuator, etc. Liquid crystal elastomer is one of the directions that deserve special attention (Lee et al., 2022; Liu G et al., 2022). The evolution of artificial muscle applications is relatively smooth and bionic arm patent applications show a declining trend. Twisted fiber (Leng et al., 2021b) and soft robot (Mirvakili et al., 2020) are the topics with more patent growth since 2020.

**TABLE 7:** Evolution of artificial muscle terms since 2018.

J-term	Time interval frequency		P-term	Time interval frequency	
	2020-2021	2018-2019		2020-2021	2018-2019
pneumatic artificial muscle	104	97	pneumatic artificial muscle	199	187
artificial muscle	72	76	artificial muscle	185	161
Actuator	40	23	actuator	76	71
soft robotics	39	39	robot	64	70
shape memory alloy	17	10	muscle	38	29
soft actuator	17	20	sensor	33	33
Muscles	16	4	fiber	31	26
Twisted fiber actuator	16	3	flexibility	29	32
Dielectric elastomer actuator	14	14	motor	29	17
carbon nanotubes	11	5	finger	26	14
hysteresis	10	2	twisted fiber	25	12
modeling	10	10	bionic arm	24	32
piezoelectric actuator	8	1	arm	23	27
robotics	8	4	joint	23	25
Smart materials	8	4	carbon nanotubes	22	21
textile fibers	8	4	soft robot	22	7
control	7	4	temperature	21	15
Force	7	3	textile fabric	21	22
liquid crystal elastomer	7	2	electrode	20	21
sliding mode control	7	8	low cost	17	15

Note: J-term = author keywords of papers; P-term = abstract NLP, of patents.

## 4 Discussion

The scientific and technological innovation of artificial muscles has a long history, but the real rise did not start until 20 years ago. Since 2000, there has been a great deal of interest and invest of science and technical issues in artificial muscles. It is still far from a large scale application of artificial muscles. Researchers are enthusiastically devoting themselves to the research of artificial muscles and are achieving an increasing number of results.

### 4.1 Rapid development and shifting focus

There are high expectations for artificial muscles. The research shows that many institutions, especially enterprises, have started the patent layout of artificial muscles early, and research results will be applied for patent protection as soon as they are considered to have the potential to be transformed into practical applications. From the characteristics of the achievements, papers regarding artificial muscles strive to achieve innovation in materials and methods, and constantly open up new horizons in artificial muscle research, while patents pay attention to cost, effectiveness and function, so that artificial muscles can be applied to people’s lives and industrial production as soon as possible.

The rapid expansion of the scale of scientific and technological innovation achievements is a perfect proof of the rapid development of artificial muscle technology. From 2000 to 2021, the number of research papers and patents on artificial muscle grew from 13 to 10 to 191 and 433, respectively. In the past 20 years, more than 5,000 researchers from over 1,000 institutions have been involved in the research on artificial muscles, with the research results being distributed in 492 academic journals. Artificial muscle research has covered the areas of materials science, robotics, instruments and instrumentation, electrical and electronic engineering, involving nearly one hundred categories. In terms of technological innovation, materials science, mechanics and automation, and robotics are the triumvirate driving the artificial muscle technological innovation, with the participation of dozens of fields such as textile science, surgery, and aeronautics.

We can also find that the center of artificial muscle science and technological innovation is migrating to emerging countries, especially to universities in emerging countries. The United States, Japan, Germany and other developed countries are the pioneers of artificial muscle research and development, but the global scientific and technological innovation center is migrating to emerging countries/regions. Research institutions and scholars from China, South Korea, India, and Iran have grown to be a significant force in artificial muscle R&D. However, countries such as the United States and Japan still have enormous influence and maintain the lead in terms of citations of papers and the attractiveness of research institutions. In terms of technological innovation, the current applications of artificial muscles are mainly in the field of health and medical devices, but the involvement of companies is modest. As for basic research, research papers are mainly from university researchers. For application development, artificial muscle patent applicants are also increasingly being from universities rather than companies.

### 4.2 Soft actuators and robots have been the focus

Robots have entered the manufacturing and daily life of people from science fiction. Autonomous robots include drive, energy, sensory and control systems that mimic various systems containing high complexity in animals and other living organisms (Aubin et al., 2022). With the refinement and miniaturization of autonomous robot design and scale, soft actuators are gradually replacing traditional actuators to provide higher integration and energy density for robots. The advent of artificial muscles has allowed soft actuators to better mimic biological muscles, adapt to their surroundings, and collaborate with the various components of the robots (Zou et al., 2021). Artificial muscles made of elastomeric dielectric materials can deform in response to light, heat, electricity, and magnetic stimuli to actuate soft robots. The use of soft materials allows soft robots to be lighter than conventional robots and to work safely with humans, as well as to adapt to different shapes autonomously.

Nowadays, artificial muscles are the core components of intelligent and interactive soft robotics systems. Researchers even proposed the concept of “artificial super muscles” to give artificial muscles better properties for more complex human-computer interaction and integration drives (Higueras-Ruiz et al., 2022). In the last 2 years, the performance metrics of artificial muscles have been upgraded and have continued to gain new applications in the field of soft robotics. We can see the refinement of artificial muscle powered insect-sized robots (Wang Z et al., 2021; Kim D et al., 2022), the improvement of artificial muscle driven water-walking robots (Zhou X et al., 2021; Kim S et al., 2022), soft tension robots for exploring unknown spaces (Kobayashi et al., 2022; Zhou et al., 2022a), the enhancement of artificial muscle driven soft crawling robots (Liu et al., 2021; Wu et al., 2022), the realization of rehabilitation assistance training robot based on pneumatic artificial muscles (Wang and Xu, 2021; Chu et al., 2022; Tsai and Chiang, 2022), control optimization and modeling of artificial muscle-actuated endo-exoskeleton robots (Chen et al., 2022; Lin et al., 2021; Liu H et al., 2022; Yang et al., 2022), and the application of SNA in soft wearable robots (Jeong et al., 2022).

Soft robots also occupy an influential position in the technological invention of artificial muscles. As seen from the highly cited patents, the citation frequency of medical robot patents such as artificial skeletal mechanism for robotic actuator, wearing hand function rehabilitation training robot is rising rapidly over time. Emerging artificial muscle patents are mainly on robot device and soft robot. The latest patents on versatility, performance, and reliability improved artificial muscles, artificial muscle lock mechanism, cut-off valves, and liquid pressure control device of the robot device are highlighted by companies, universities and research institutions.

### 4.3 Emergence of smart materials

Artificial muscles are sometimes considered to be and equivalent to smart muscles, but not all artificial muscle types actually belong to the category of smart materials. A variety of artificial muscles have been manufactured from metals, polymers, ceramics, and diverse composite materials, many of which do not have intelligent components. The familiar PAMs are non-smart muscles that do not contain any smart materials. Similarly, hydraulic artificial muscles are not smart, but as one of the most mature artificial muscle technologies, they are widely used, and organizations such as Soft Robotics Inc. and Soft Gripping have patented and manufactured a variety of soft robots. Research on various artificial muscles made of smart materials, such as CNTs and SMAs, has been extremely active in recent years.

PAMs remain one of the most widely used artificial muscles. In recent years, researchers have made new progress in the direction of three-dimensional printing miniature piezoelectric actuators (Cui et al., 2019), energy harvesting and sensing soft piezoelectric materials, capacitive wearable haptic sensing devices (Ozioko et al., 2020), humanoid arm robots actuated by PAMs (Liang et al., 2022b), multi-directional bending PAMs (Xiao et al., 2021a; Xiao et al., 2021b), amplified piezoelectric actuators based on piezoelectric hydraulic pumps (Park and Cha, 2019), origami micro-manipulators for minimally invasive surgery (Suzuki and Wood, 2020), and non-tethered flapping wing micro-vehicles (Shi et al., 2020). The number of patents for artificial intelligence and soft robots based on PAMs is also growing rapidly.

PAMs, hydraulic artificial muscles, and other non-smart artificial muscles have enabled soft actuators with better mechanical properties to be fully integrated with various electronic devices to make soft actuators and robots more intelligent, a new trend in research in recent years. Smart artificial muscles should have the ability to sense various external stimuli such as temperature, pressure, light, magnetism, color, sound, etc. and respond quickly and autonomously. Smart artificial muscles include DEAs, IPMCs, etc. Each smart material has its application area.

In recent years, researchers have made a lot of achievements in the scientific and technological innovation of intelligent artificial muscles. Among them, novel artificial muscles such as twisted and coiled artificial muscles (TCAMs) and alkene-carbon artificial muscles are particularly noteworthy.

TCAMs have attracted attention in the field of smart actuators and robotics due to their low time lag, high operating density, and low manufacturing difficulty. They are made of twisted and coiled polymer fibers that form a spring-like structure and can contract or expand tremendously when heated. In the past few years, researchers have reported a variety of TCAMs, including TCAMs Driven Soft Crawling Robot (Wu et al., 2022), Modeling framework for macroscopic dynamics of twisted and coiled polymer actuator driven by Joule heating focusing on energy and convective heat transfer (Masuya et al., 2017), force enhanced multi-twisted and coiled actuator (Zhou et al., 2022b), CN-polypyrrole coated twisted and coiled yarn artificial muscles (Aziz et al., 2020), spandex fibers and SMA Skeleton TCAMs (Zhang and Yang et al., 2022), etc. Twisted and coiled soft actuators used for special clothing, twisted and coiled carbon nanotube yarn annealing and reinforcement process, and TCAMs array devices have been applied for patents on their technologies, providing a foundation for commercial production.

Alkene-carbon materials such as CNT and graphene have outstanding advantages in terms of tensile strength, thermal conductivity, electrical conductivity and specific surface area (Plisko and Bildyukevich, 2014), and have promising prospects for applications. In recent years, alkene carbon material artificial muscles programmable response deformation (Kim et al., 2020), multi-directional actuation (Oh et al., 2021; Ling et al., 2020), integrated actuation and motion sensing (Zhao et al., 2020; Wang H Q et al., 2020), laser-induced graphene soft actuators (Ling et al., 2020), bilayer membrane structure temperature sensing actuator (Xiao Y W et al., 2021), and other directions have shown new progress, which are expected to meet the demand for more scenarios of artificial muscle applications.

Artificial muscles have not been able to achieve a full performance advantage over conventional actuators. Artificial muscles with large output stresses, such as piezoelectric actuators, are usually limited in output strain. The opposite is the case of dielectric elastomers. Some of the novel artificial muscles have achieved a better balance between stress and strain, yet face a trade-off between actuation rate and efficiency (Greco et al., 2022). Various smart materials have different drive modes, physical properties and manufacturing processes, all with pros and cons. Therefore, before the emergence of a more comprehensive performance smart actuator, it is necessary to determine the type of artificial muscle according to the application scenario so as to obtain the best environmental sensitivity and intelligent interaction capability.

### 4.4 The future of artificial muscles

The application of artificial muscles in a wide range is already visible, including some interesting frontiers such as programmable biomimetic actuators (Dong et al., 2019), shape-color-switchable thermochromic actuators (Wang X et al., 2019), space robots (Ashby et al., 2022), and micro vehicles (Chen and Zhang, 2019). All signs indicate that artificial muscles are competitive in replacing conventional actuators in areas that require strong human-machine interaction and strong adaptability to the surrounding environment now. Researchers are also looking ahead to more thrilling applications of artificial muscles, especially for more intelligent robots that adapt to complex environments. The combination of artificial muscle technology with artificial intelligence technology, wireless communication technology and novel energy storage technology is expected to allow intelligent robots to take the place of humans in scientific research activities in every extreme environment, down to the Marianas Trench, up to Mount Everest, and far to the Moon and Mars. Meanwhile, micro-robots with artificial muscle technology are highly anticipated. It is hoped that such micro-robots can autonomously complete various *in vivo* tests and treatments to relieve patients’ pain and save more lives.

Recent research results also show that although the scientific and technological innovation of artificial muscles is gaining momentum, and some of the properties of artificial muscles have surpassed those of biological muscles, no artificial muscle has completely replaced biological muscles, and there are still many issues that need to be solved. The first issue is the cost as the transformation of graphene, CNTs, SMAs, SMPs, and other artificial muscle materials into artificial muscles is still very costly, and there is still a big gap from mass production. The next issue is reliability. Temperature difference drive, solvent adsorption drive or electrochemical drive can not solve the problem of slow response speed and long recovery time of artificial muscles. In addition, the sustainability also needs to be improved, the energy conversion efficiency of existing artificial muscles is still far below that of traditional electric motors or heat engines. Researchers have proposed to improve artificial muscles by appropriately cooling the fibers, increasing Joule heat to assist adsorption, preparing non-metallic IPMC, and increasing the ion diffusion rate of electrolytes, but they have not yet been widely employed. To better reproduce natural muscle functionalities, researchers treat artificial muscle as a dynamic non-linear system and introduced various methods such as adaptive control law, adaptive fuzzy-sliding mode control method, learning-based adaptive robust control framework, etc, but there is still a lot of distance from lib to life. People are also continually updating technology and improving materials in the hope that artificial muscles can actuate soft robots better with programmable motions (gripping, twisting, and three-dimensional bending) (Nuchkrua and Leephakpreeda, 2022) in the near future. The future research and development of artificial muscles still needs to strengthen the cross-fertilization of subjects such as material science, computer science, machinery and automation, improve the synergy of enterprises, universities, and research institutions, continuously upgrade materials, improve performance, and reduce costs.

According to the needs of production and life of human beings, the target of artificial muscle material improvement and technology enhancement should be to improve the response speed, enhance cycle stability and significantly reduce manufacturing costs to achieve billions of drive cycle mechanical applications and meet the needs of billions of people worldwide for soft robots, medical devices, and kinds of artificial intelligence applications.

## 5 Conclusion

Studies of artificial muscles represent a rapidly growing field. This paper uses bibliometric methods to provide a brief review of the development of artificial muscles based on papers and patent data, retrospect and analyze the progress and status of artificial muscles based on SMAs, CNTs, graphene, and other materials, and prospects the future development of artificial muscles. This paper will help researchers to grasp the overall picture of artificial muscle research, follow the frontiers of scientific and technological innovation, determine research directions and select journals for submission, and help enterprises in patent layout.

There are still some limitations in this study. It is a reasonable and common research method to use papers to refer to scientific research and patents to refer to technological innovation (Jin et al., 2023), but patents need to be transformed into products to complete R&D even patents covered more than 90% of the latest technological information in the world (Milanez et al., 2014), so there are different views. The bibliometric method can help researchers to get a comprehensive picture of a scientific issue, but biases can occur for some reasons. For this study, the pre-defined retrieval formula and the restricted retrieval field cannot cover the artificial muscles papers and patents completely and accurately, although we carefully designed the retrieval process according to the specifications and introduced human intervention to remedy this.
